# Reconstruction of sternal defects after sternotomy with postoperative osteomyelitis, using a unilateral pectoralis major advancement muscle flap

**DOI:** 10.1038/s41598-020-65398-y

**Published:** 2020-05-20

**Authors:** Alexander Wyckman, Islam Abdelrahman, Ingrid Steinvall, Johann Zdolsek, Hans Granfeldt, Folke Sjöberg, Hans Nettelblad, Moustafa Elmasry

**Affiliations:** 1 Department of Hand Surgery, Plastic Surgery and Burns, and Department of Biomedical and Clinical Sciences, Linköping University, Linköping, Sweden; 2 Plastic Surgery Unit, Surgery Department, Suez Canal University, Ismailia, Egypt; 3 Department of Thoracic and Vascular Surgery in Östergötland, and Department of Health, Medicine and Caring Sciences, Linköping University, Linköping, Sweden

**Keywords:** Outcomes research, Infectious diseases, Muscle, Infection, Diseases, Medical research, Risk factors, Signs and symptoms

## Abstract

Background: The pectoralis major flap, which is usually harvested bilaterally, is considered a workhorse flap in the reconstruction of sternal defects. After a median sternotomy for open heart surgery, 1%-3% of patients develop deep infection and dehiscence of the sternal wound, some of which will eventually require reconstructive surgery. Our aim was to describe the clinical feasibility and associated complications of the unilateral pectoralis major advancement flap in the reconstruction of sternal defects. Methods: A retrospective analysis of all adult patients who were operated on using a unilateral pectoralis major flap for reconstruction of the chest wall at the Linköping University Hospital during 2008–18 was made using data retrieved from medical records. Results: Forty-three patients had reconstructions with unilateral pectoralis major flaps. Three flaps failed completely, and another 10 patients developed complications that required further operation. The factors that were independently associated with loss of the flaps and complications were: older age, male sex, the number of different antibiotics used, and a long duration of treatment with negative wound pressure. Fewer wound revisions before the reconstruction resulted in more complications. The factors that were independently associated with prolonged time to complete healing were emergency reoperation after the initial operation and complications after reconstruction. Conclusion: The unilateral pectoralis major advancement flap has proved to be a useful technique in the reconstruction of most sternal defects after sternal wound infection in older patients. There is, however, need for a follow-up study on a larger number of procedures to evaluate the long-term outcome compared with other methods of sternal reconstruction.

## Introduction

Median sternotomy is one of the most commonly used incisions in open heart surgery. After it, 1–3% of patients develop deep sternal wound infections^1^ according to the CDC guidelines^2,3^. Factors such as old age, coexisting medical conditions (such as diabetes, chronic obstructive pulmonary disease, high body mass index (BMI), and hypertension) and the harvest of the internal mammary artery (IMA) can lead to a deep sternal wound infection^3^.

Sternal wound dehiscence can be managed conservatively by regular wound dressings, application of negative pressure to the wound, debridement, and rewiring of the sternum. If this fails, a local muscle flap is recommended to reconstruct the defect. The flap can also be used to reconstruct the defect directly after a thorough wound revision^1,4,5^.

There are various local flaps used in the reconstruction of sternal defects, of which the pectoralis major muscle flap is considered a workhorse flap. Others include omental flaps, rectus abdominis flaps, and latissimus dorsi flaps. Another option is to use a free flap such as the anterolateral thigh or gracilis muscle flap, but the absence of recipient vessels as a result of their harvest during the primary cardiovascular operation is a limitation^6,7^.

The pectoralis major muscle lies on the anterior upper chest, originates from the sternum and clavicle, and inserts on the superolateral bicipital groove of the humerus. Different flap reconstruction techniques utilizing it have been described such as bilateral advancement, unilateral advancement combined with a turnover flap on the other side, a unilateral turnover flap, and unilateral advancement rotation.

Each of these techniques has its advantages and limitations. For example, turnover flaps always require an intact IMA with medial perforators to supply the muscle. Additionally, the flap may be jeopardised in case of emergency re-entry into the chest^8^. It has been argued that the unilateral flap could be inferior to the bilateral flap in its ability to cover the whole defect, and affect the long-term outcome^9^.

In our department we have considerable experience with the unilateral pectoralis major muscle advancement flap, which has been used to cover most of the sternal defects that have presented to our reconstructive surgical team. To the best of our knowledge, there are relatively few reports that focus solely on this surgical approach^10–13^.

The aim of our study was to describe outcomes after the reconstruction of sternal defects using a unilateral advancement rotational pectoralis major flap, to establish its clinical feasibility, and document the associated complications.

## Methods

**Figure 1:**
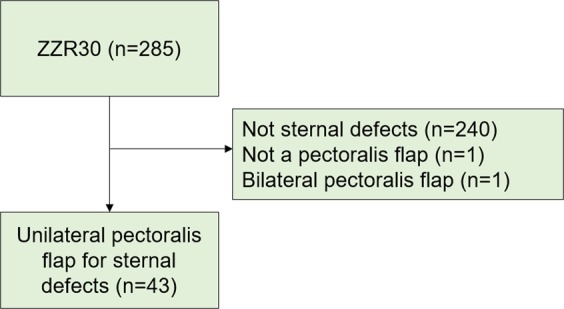
Flowchart showing the selection of patients. ZZR30 is the ICD-10-SE procedure code for “reconstruction with a muscle flap”. A total of 45 reconstructions of sternal defects were done with muscle flap, 43 with a unilateral pectoralis muscle flap, one with bilateral pectoralis muscle flaps and one with another muscle flap.

**Figure 2:**
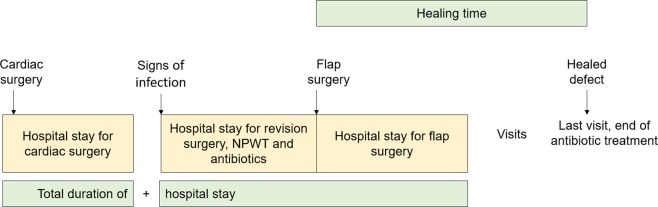
Flowchart showing different times of admissions for patients who had open heart surgery and were later readmitted for revision surgery, treatment using negative wound pressure, and antibiotics, when they developed signs of sternal instability or sternal infection, or both. If reconstructive surgery was indicated, the necessary flap was revised. The last visit, when the antibiotic treatment could be ended, was classed as the end point for healing of the defect. NPWT = negative pressure wound therapy.

All adult patients who had had procedure ICD-10-SE code ZZR30 (reconstruction with a muscle flap) for sternal defects during 2008–2018 by plastic surgeons from the Department of Hand and Plastic Surgery at Linköping University Hospital were screened. All patients who had had reconstructions with a unilateral pectoralis major flap were included (Fig. 1), and a retrospective analysis was made using data retrieved from their medical records. The following variables were used: age, sex, BMI, coexisting medical conditions, variables related to the operation, indication for flap surgery, antibiotic treatment, results of microbiological culture, viability of the flap, and complications, duration of hospital stay for cardiac surgery and for flap surgery, healing time (defined as the last day of antibiotic treatment), and donor-site complications. Figure 2 shows a flowchart of the different time periods.

### Care after cardiac surgery

The patients included were initially admitted to the department of cardiothoracic surgery in Linköping University Hospital, and most of them were then discharged. Those who developed signs of sternal instability or sternal infection, or both^3^, were readmitted to the department of cardiothoracic surgery for revision surgery, and treatment with negative wound pressure and antibiotics. Some patients were operated with sternal rewiring and/or plating, and sternal resection by the thoracic surgeons. If flap surgery was still needed, a plastic surgeon was consulted.

### Operative technique

**Figure 3:**
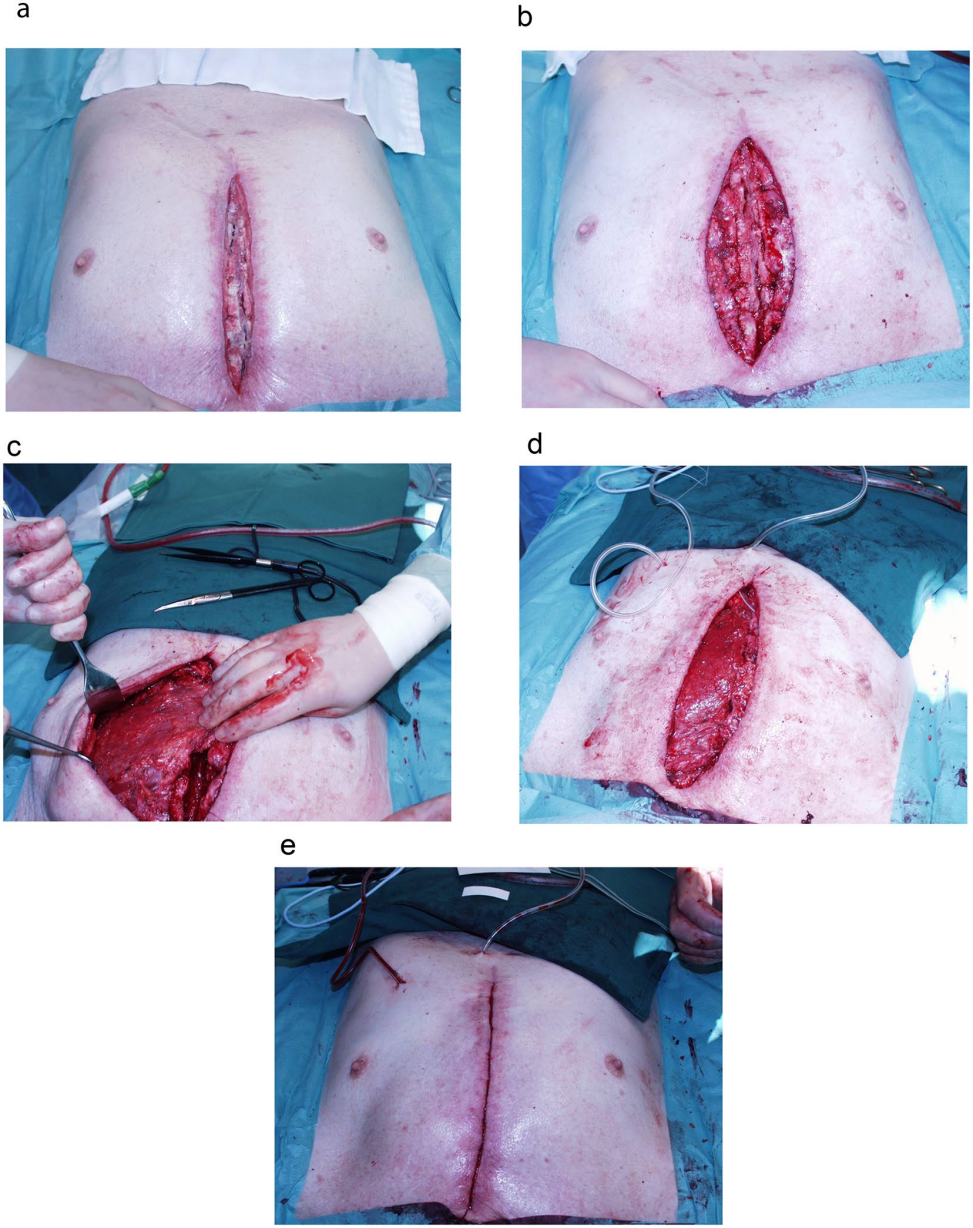
(**A**) Sternal wound with deep infection after cardiac surgery. (**B**). Sternal wound after debridement. (**C**) The left pectoralis major muscle being mobilized after dissection. (**D**) The pectoral flap sutured in place to cover the defect. (**E**) After skin closure.

All pectoralis major muscle flaps were operated on by plastic surgeons using a standard technique for unilateral advancement rotation, which allowed the harvest of enough unilateral pectoral muscle tissue to cover the entire sternal defect. Figure 3A–E shows various steps of the operation. The dissection started from the border of the median sternotomy along the superficial side of the muscle (separating skin and subcutaneous tissue from the muscle) to the diagonal lateral border of the muscle. This is most easily identified cranially, where the lateral edge is a rather thick roll, slightly elevated from the thoracic cage. After identification of the lateral border, dissection proceeded caudally until the most cranial part of the ipsilateral rectus abdominis muscle was reached. Meticulous separation of the muscular origin from the clavicle, carefully preserving the thoraco-acromial vessels, in addition to complete transection of all insertions of the pectoral muscle to the humerus, permits a generous range of advancement-rotation of the muscle, allowing insertion of the medial edge of the muscle into the inter-sternal defect by way of a 90 degree fold of the muscle´s anterior border as illustrated in Fig. 3D. Coverage of the defect was performed by the remaining muscle, secured in place with absorbable sutures to the right sternal remnant. The undermined skin flaps on both sides were closed in the midline. Figure 3E shows the obliquely running ridge across the left thorax. This is a demarcation of the new position of the lateral border of the pectoral muscle and illustrates the range of advancement of the muscle that can be attained by the extensive release of the muscle.

**Figure 4:**
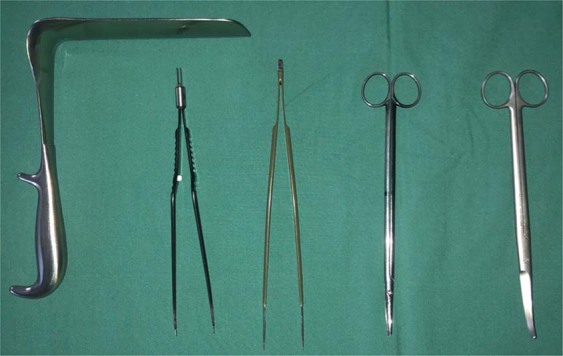
A set of special long surgical instruments: long retractor, bipolar diathermy forceps, tweezers, straight and curved scissors.

The extensive dissection and liberation of the pectoral muscle is greatly simplified by the use of a small set of special long surgical instruments (Fig. 4). This technique allows the full dissection of the muscle while avoiding an extra incision in the anterior axillary line. The use of unilateral pectoralis major saves the patient the co-morbidity associated with bilateral harvesting of the muscle.

### Statistics

Data are presented as median (IQR) unless otherwise stated. Normality of distribution was tested with the Lilliefors test. Mann Whitney U and the chi-squared tests were used to test differences between groups. Statistical significance was set at p < 0.05. Logistic regression was used to assess the significance of differences between the effects of age, sex, BMI, number of antibiotics, variables related to cardiac surgery, and presence of coexisting medical conditions on outcome (complications, including flap loss, were coded as 1). Linear regression was used to assess the effects of age, sex, BMI, number of antibiotics, variables related to cardiac surgery, and the presence of coexisting medical conditions on duration of healing.

### Ethics

All methods in this study were carried out in accordance with relevant guidelines and regulations. The study was approved by the Regional Ethics Review Board in Linköping, Sweden.

As the study design was retrospective no experimental protocols were used. No subjects under 18 were included.

The figures presented in this manuscript cannot reveal patient’s identity and the study design was retrospective. Additionally, the results were not presented in the individual level. The Regional Ethics Review Board for these reasons did not mandate individual consents from the patients included in the study.

## Results

### General results

A total of 43 patients had unilateral pectoralis major muscle flaps inserted, of which 38 (88%) were on the left. In one of the patients the flap was placed directly after resection of the sternum as part of reconstruction of the chest wall. Median age was 76.2 (IQR: 69.4–80.1), three patients died, and in another three patients the flaps failed (Table 1).

**Table 1:** Description of the patients.

Number of patients	43
Age (at flap surgery)	76.2 (69.4–80.1)
BMI	27.9 (24.8–31.4)
Co-existing medical conditions	
Cardiovascular disease	41 (95)
Diabetes	13 (30)
Malignancy	7 (16)
Renal failure	4 (9)
Corticosteroid treatment	7 (16)
Chronic obstructive pulmonary disease	5 (12)
Other diagnoses	22 (51)
Smokers	4 (9)
Former smokers	14 (33)
Non-smokers	25 (58)
Operation time, minutes	113 (86–139)
Anaesthesia time, minutes	165 (140–195)
Duration of stay for flap surgery, days	12.4 (7.3–18.0)
Total duration of stay, days^a^	39.0 (30.0–50.0)
Time between thoracic surgery and flap surgery, days	35.6 (21.7–50.4)
Mortality during hospital stay	3 (7)
Left-sided pectoralis major flap	38 (88)
Female sex	14 (33)

Data are presented as median (25–75 centile) or n (%).

^a^Duration of stay during thoracic surgery and flap surgery.

### Results of cardiac surgery

The different procedures included coronary artery bypass grafting, and mitral and aortic valve replacement or repair. Some of the patients had combined procedures. The vessel used as the graft in coronary artery bypass grafting was the left IMA in most procedures. A total of six patients required emergency reoperation as a result of postoperative tamponade. The median (range) duration of stay was 10 (7–14) days in the cardiothoracic surgery department (Supplemental Table 1).

After sternal infection had been diagnosed patients were re-admitted for wound debridement. The median hospital stay for revision surgery was 14 days and nearly all were treated with negative wound pressure. The patients had a median of four surgical debridements before a pectoralis major muscle flap was used.

### Flap results

The median duration of stay after flap surgery was 12 days. Of the 43 pectoral flaps, three patients were not included in the analysis of flap failure as they died shortly after the operation before viability could be assessed. A total of 37 flaps were considered to be successful and three had failed and required further reconstruction.

Complications in the failure group were wound dehiscence, recurrent infection, fistula formation, and necrosis of the skin or flap. These patients required more reoperations and had longer stays in hospital than those in the successful group (Table 2).

**Table 2:** The difference between the patients grouped by flap outcome.

	Flap failure (n = 3)	Flap success (n = 37)	P-values
Patient factors			
Age (at flap surgery)	78.6 (76.2–83.5)	74.4 (68.2–80.1)	0.32
BMI	31.1 (28.1–38.9)	27.8 (24.6–31.4)	0.18
Female sex	0	12	0.54
Pre-existing medical conditions			
Diabetes	1	10	1.00
Smoking	3 (100)	15 (41)	0.08
COPD	0	5	1.00
Cardiovascular disease	3	35	1.00
Corticosteroid treatment	1	6	0.45
Other	3	17	0.23
Malignancy	0	7	1.00
Renal failure	0	2	1.00
Number of patients with complications	3	16	0.10
Number of patients with complications not requiring surgery	0	7	1.00
Types of complications not requiring surgery			
Seroma or hematoma	0	7	1.00
Fistulation	0	1	1.00
Number of patients with complications requiring surgery	3	9	0.02
Types of complications requiring surgery			
Post-operative bleeding	2	7	0.12
Signs of persistent infection	1	3	0.28
Wound dehiscence	0	3	1.00
Fistulation	0	2	1.00
Skin necrosis	2	0	0.04
Flap detachment	1	0	0.08
Patients reporting pain/discomfort in flap area at follow-up visits	0	9	1.00
Duration of stay (flap)	31 (15.6–89.3)	11.4 (7.3–16.6)	0.02
Total duration of stay	50 (48–122)	38 (30–47)	0.048
Operation time	150 (75–155)	110 (86–135)	0.46
Anaesthesia time	210 (115–235)	163 (140–195)	0.29

Data are presented as median (25–75 centile) or n.

Apart from the flap failure group, 10 patients required reoperation for complications, although eventually they all healed completely. The most common complications that required reoperation were large postoperative haematomas and wound infections that required debridement. The most common complications that did not require reoperation were seroma and small haematomas (Table 3).

**Table 3:** Flap outcome and complications.

Viable flap^a^	37 (93)
Non-viable flap^a^	3 (8)
Healing time^b^	47.4 (27.6–60.6)
Number of patients with complications	21 (49)
Number of patients with complications not requiring surgery	8 (19)
Types of complications not requiring surgery	
Seroma or hematoma	8
Fistulation	1
Number of patients with complications requiring surgery	13 (30)
Types of complications requiring surgery^c^	
Post-operative bleeding	10
Signs of persistent infection	4
Wound dehiscence	3
Fistulation	2
Skin necrosis	2
Flap detachment	1
Patients reporting pain/discomfort in flap area at follow-up visits	9

^a^The calculation of percentage is done by using 40 patients as three patients died within 30 days of the flap surgery.

^b^The time between flap surgery and the last day of antibiotic treatment, calculated on the group with viable flaps.

^c^One patient could have several complications contributing to the decision of re-operation.

Forty-two patients were treated with antibiotics after flap surgery and 38 had at least one positive microbiological culture. The most common bacteria found in microbiological cultures from the sternal wound were Coagulase-negative staphylococci, *Propionebacterium acnes* and *Staphylococcus aureus*. Growths of *Candida albicans*, *Enterobacter cloacae*, and *Klebsiella variicola* were more common in the flap failure group (Supplemental Table 2).

The most commonly used antibiotics were vancomycin, clindamycin, and linezolid. The patients in the failed flap group were treated with more different antibiotics than those in the successful group (Supplemental Table 3).

### Multivariable regression

The factors that were independently associated with complications, such as flap loss, were older age, male sex, the total number of different antibiotics, the number of days using negative wound pressure treatments, and fewer revisions (Table 4). The factors that were independently associated with healing time were reoperation after the cardiac surgery and complications after flap surgery, including loss of the flap (Table 5).

**Table 4:** Logistic regression for flap loss and complications.

	Coefficient	p	95% CI
Age, years	0.25	0.04	0.02 to 0.49
Body mass index	−0.17	0.20	−0.43 to 0.09
Female sex, male is reference	−8.80	0.02	−16.17 to −1.43
Number of revisions/ NPWT dressing changes	−2.39	0.04	−4.64 to −0.15
Total number of different antibiotics	1.95	0.03	0.20 to 3.69
NPWT-treatment prior to flap surgery, days	0.79	0.04	0.05 to 1.53
Constant	−18.93	0.07	−39.51 to 1.64

Multivariable logistic regression pseudo R^2^ 0.58, Model p < 0.001, n = 41. Flap loss and complications (n = 21) are coded as 1. Flap success with no complications are coded as 0. NPWT = Negative pressure wound therapy.

**Table 5:** Linear regression for healing time.

	Coefficient	p	95% CI
Re-operation, thorax	46.00	0.003	17.17 to 74.77
Flap loss and complications, presence	33.38	<0.001	16.03 to 50.73
Female sex, male is reference	5.16	0.59	−14.37 to 24.70
Body mass index	1.28	0.19	−0.67 to 3.23
Age, years	−0.52	0.24	−1.41 to 0.37
Constant	29.69	0.50	−59.19 to 118.56

Multivariable linear regression R^2^ 0.54, model p < 0.001, n = 35.

## Discussion

Forty-three patients with complex sternal defects had reconstructions with unilateral pectoralis muscle flaps. There were a total of 37/40 successful flaps in this older group of patients (median 76 years of age) with multiple coexisting conditions such as cardiovascular disease and deep sternal wound infections.

One of the most commonly used methods of reconstruction is the bilateral pectoral muscle flap, which has been shown to lead to better sternal stabilisation than the rewiring of the sternum without a muscle flap^14^. It has been discussed whether a unilateral pectoral muscle flap can suffice to reconstruct the sternum and successfully resolve deep sternal wound infections in adults. Fernandez-Palacios et al. compared 19 unilateral and 13 bilateral pectoral flaps and found that the unilateral technique was significantly associated with a shorter operating time; less need for blood transfusions postoperatively; and earlier extubation. Both methods had similar results regarding mortality, morbidity, and complications^12^. Horácio et al. showed good results after the use of unilateral pectoral muscle flaps in their study of 11 patients^13^. The main advantage of the unilateral pectoral muscle flap is that it preserves full strength in at least one arm, and the possibility of saving the contralateral pectoralis major in case of possible flap failure.

There are many different techniques that can be used to mobilise the pectoralis muscle flap, which can be used with either a single unilateral flap or bilateral flaps. Zahiri *et al*.^15^ recommended the turnover technique over the advancement technique, as the former has fewer complications. However, this requires the presence of at least one IMA, and even if one is still present there may be a risk of inadequate blood supply to the flap, which could lead to necrosis and flap failure. The advancement technique is considered a safer option, because the blood supply of the flap is then based on the thoracoacromial pedicle. Berg et al. recommended the unilateral turnover split technique for greater coverage of the whole sternal wound^16^. The advancement flap offers excellent coverage of the superior portion of the sternum, but it has been noted that it is insufficient to cover distal sternal wounds, particularly if the humeral insertion of the muscle has not been detached^4,17,18^.

The post-operative mortality (7%) and morbidity (3 with flap failure and 10 with re-operations) numbers are relatively high in the present study. This can be explained by the advanced age of the patients as well as a high degree of cardiovascular comorbidities, which are prominent risk factors for deep sternal wound infection^3^. However, the rate of complications is still in line with previous reports^13,16,18^. Postoperative bleeding was the most common cause of reoperation (Table 3). This is in line with other studies that have shown an increased risk of postoperative bleeding if the humeral insertion of the pectoralis muscle was detached during operation, which was the case in our study^18^. The choice of technique, as well as attempts to shorten the operating time, should be taken into consideration to achieve the best results for this susceptible group.

The definition of healing time in this study has not been used often but it was used as a way to standardise the measurement of healing time in the present study. Antibiotic treatment was stopped after clinical examination of the patient at follow up together with an infectious disease specialist, if no signs of infection were evident. Regarding antibiotic treatment we found that more different antibiotics per patient were associated with failure of the flap (Table 4).

More sternal revisions were associated with better outcomes in our study, which may have been the result of a more thorough revision which stimulates the formation of granulation tissue. Some authors have recommended early sternal reconstruction with a pectoral muscle flap together with surgical debridement of the sternum in a single stage, as it would result in a shorter duration of hospital stay and less time in the ICU after the operation^4,5^. However, other studies show that delayed closure of the wound with muscle flaps, when systemic infection has subsided, decreases the rate of wound complications compared with early closure^19^.

Others have shown considerable benefits from the use of negative pressure wound therapy (NPWT) compared with conventional wound care^1,6,7,20,21^, and a few studies have even reported success with treating sternal dehiscence using it solely^20,22^. In our study most patients were treated with both NPWT and several sternal revisions prior to flap surgery. Our results may be of importance in the assessment of guidelines regarding the treatment of deep sternal wound infections.

### Limitations

This study is a single centre retrospective study with a relatively small study group, which may affect the generalisability of the results. However, compared with other studies on the unilateral pectoral muscle flap, our study group was representative.

As we relied on data from patients’ medical records, there was a risk of the over-representation of negative findings such as complications, because these patients would probably have generated more visits and journal notes.

We were not able to retrieve data about the size of the sternal defects, which would have been interesting to show the maximum size of the defects that we could cover using this technique.

Our long-term results and findings on donor-site morbidity are derived solely from patients’ medical records, and because of this we had to rely on the documentation of the physician present at the follow-up visit. Follow up was mainly handled by the cardiac surgeons, who might not have focused on the plastic surgery perspective, and because of this, long-term follow up is needed to evaluate these variables further.

## Conclusions

The unilateral pectoralis major advancement flap has proved to be a useful technique in the reconstruction of most sternal defects after sternal wound infection in older patients. There is, however, need for a follow-up study on a larger number of procedures to evaluate the long-term outcome compared with other methods of sternal reconstruction.

## Supplementary information

Supplementary information.
