# Supplementary material for: Reconstruction of sternal defects after sternotomy with postoperative osteomyelitis, using a unilateral pectoralis major advancement muscle flap
Source: Sci Rep. 2020 May 20;10:8380. doi: 10.1038/s41598-020-65398-y (PMC7239941; doi:10.1038/s41598-020-65398-y)
Supplement: Supplementary file 1 — Supplementary information. [file 41598_2020_65398_MOESM1_ESM.docx]

# Reconstruction of sternal defects after sternotomy with postoperative osteomyelitis, using a unilateral pectoralis major advancement muscle flap

Alexander Wyckman MD^1^, Islam Abdelrahman MRCS, FEBOPRAS, MD, PhD^1,2^, Ingrid Steinvall PhD^1^, Johann Zdolsek MD, PhD^1^, Hans Granfeldt MD, PhD^3^, Folke Sjöberg MD, PhD^1^, Hans Nettelblad MD, PhD^1^, Moustafa Elmasry MD, PhD ^1,2^

**Affiliations**

^1^Department of Hand Surgery, Plastic Surgery and Burns, and Department of Biomedical and Clinical Sciences, Linköping University, Linköping, Sweden

^2^Plastic Surgery Unit, Surgery Department, Suez Canal University, Ismailia, Egypt

^3^Department of Thoracic and Vascular Surgery in Östergötland, and Department of Health, Medicine and Caring Sciences, Linköping University, Linköping, Sweden

**Corresponding author:**

Alexander Wyckman The Burn Centre, Linköping University Hospital

Linköping, SE-58185, Sweden

Alexander.Wyckman@regionostergotland.se

Phone: +46 (0)70 261 9009

Fax: +46 (0)101033705

**Disclosure:** There was no conflict of interest associated with this study. This work was supported by, and done, at the Department of Hand Surgery, Plastic Surgery and Burns, and the Linköping University, Linköping, Sweden. No other funding was received.

**Supplemental Tables**

| **Supplemental Table 1 – Cardiothoracic surgery factors** | |  |  |  |
| --- | --- | --- | --- | --- |
|  | Total | Flap failure | Viable flap | p |
| Hospital stay for thoracic surgery | 10.0 (7.0-14.0) | 11 (7-23) | 9 (6-14) | 0.52 |
| Type of thoracic surgery |  |  |  |  |
| - Coronary artery bypass grafting | 24 | 2 | 20 | 1.00 |
| - Valve replacement/repair | 6 | 1 | 5 | 0.39 |
| - Combination | 9 | 0 | 8 | 1.00 |
| - Other | 4 | 0 | 4 | 1.00 |
| Graft vessels used in CABG |  |  |  |  |
| - LIMA | 29 | 2 | 25 | 0.85 |
| - LIMA + RIMA | 1 | 0 | 1 |  |
| - Only vein graft | 3 | 0 | 3 |  |
| Operation time (min) | 215 (175-250) | 200 (170-220) | 212.5 (175-295) | 0.61 |
| Aortic cross-clamp time (min) | 60 (46-105) | 50 (40-105) | 62.5 (48-92) | 0.53 |
| Cardiopulmonary bypass time (min) | 96 (81-156) | 90 (62-138) | 100.5 (83-172) | 0.49 |
| Re-operation, acute | 6 | 0 | 5 | 1.00 |
| Hospital stay for revision surgery | 14 (10-19.0) | 10 (8-25) | 14 (10-19) | 0.59 |
| Number of patients treated with NPWT | 42 (98) | 3 | 36 | 1.00 |
| Days with NPWT before flap surgery | 13.3 (9.6-19.4) | 9.7 (8.7-25.4) | 13.0 (9.5-19.4) | 0.90 |
| Sternal revisions/NPWT-changes^1^ | 4.0 (3.0-7.0) | 5 (2-9) | 4 (3-6) | 0.82 |
| Time between thoracic surgery and flap | 36.5 (21.7-50.4) | 25.7 (14.7-36.4) | 36 (21.7-56.4) | 0.32 |
| Data are presented as median (25-75 centile) or n (%). CABG = Coronary artery bypass grafting. LIMA and RIMA = left and right internal mammary artery. NPWT = Negative pressure wound therapy.  ^1^During the period between first thoracic surgery and flap surgery. | | | | |

| **Supplemental Table 2 – Microbiological species before and after flap surgery** | | | | |
| --- | --- | --- | --- | --- |
|  | All | Flap failure (n=3) | Viable flap (n=37) | p-values |
| Patients with positive bacteriological culture | 38 (88) | 3 | 32 | 1.00 |
| Patients with negative bacteriological culture | 5 (12) | 0 | 5 | 1.00 |
| Coagulase-negative staphylococci | 34 | 3 | 28 | 1.00 |
| Propionebacterium acnes | 13 | 0 | 12 | 0.54 |
| Staphylococcus aureus | 8 | 1 | 7 | 0.50 |
| Candida albicans | 6 | 2 | 3 | 0.04 |
| Enterococcus faecium | 3 | 0 | 3 | 1.00 |
| Enterobacter cloacae | 3 | 2 | 1 | 0.01 |
| Enterococcus faecalis | 2 | 0 | 1 | 1.00 |
| Klebsiella variicola | 2 | 2 | 0 | 0.004 |
| Klebsiella pneumoniae | 1 | 0 | 1 | 1.00 |
| Alpha haemolytic streptococci | 1 | 0 | 1 | 1.00 |
| Candida glabrata | 1 | 0 | 1 | 1.00 |
| Escherichia coli | 1 | 0 | 1 | 1.00 |
| Number of different bacteria per patient | 1 (1-2) | 4 (1-5) | 1 (1-2) | 0.14 |
| Patients who died within 30 days of flap surgery were not divided into either of the groups. | | | | |

| **Supplemental Table 3 – Antibiotic treatment post flap surgery** | | | | |
| --- | --- | --- | --- | --- |
|  | All | Flap failure | Viable flap | p-values |
| Number of patients treated with antibiotics | 42 | 3 | 36 | 1.00 |
| Vancomycin | 33 | 3 | 27 | 0.56 |
| Clindamycin | 17 | 2 | 14 | 0.55 |
| Linezolid | 16 | 1 | 15 | 1.00 |
| Cefotaxime | 14 | 2 | 12 | 0.28 |
| Rifampicin | 11 | 1 | 9 | 1.00 |
| Piperacillin/tazobactam | 11 | 2 | 8 | 0.15 |
| Fusidic acid | 9 | 0 | 9 | 1.00 |
| Meropenem | 7 | 2 | 4 | 0.05 |
| Flucloxacillin | 5 | 1 | 4 | 0.34 |
| Anidulafungin | 5 | 1 | 2 | 0.21 |
| Fluconazol | 5 | 0 | 4 | 1.00 |
| Ciprofloxacin | 4 | 0 | 3 | 1.00 |
| Cloxacillin | 4 | 1 | 3 | 0.24 |
| Trimethoprim/sulfamethoxazole | 3 | 1 | 1 | 0.15 |
| Tygecycline | 3 | 0 | 3 | 1.00 |
| Moxifloxacin | 3 | 0 | 3 | 1.00 |
| Imipenem/cilastatin | 2 | 0 | 2 | 1.00 |
| Benzylpenicillin | 2 | 0 | 2 | 1.00 |
| Cefadroxil | 1 | 0 | 1 | 1.00 |
| Caspofungin | 1 | 1 | 0 | 0.08 |
| Amphotericin B | 1 | 0 | 1 | 1.00 |
| Amoxicillin/clavulanic acid | 1 | 0 | 1 | 1.00 |
| Number of different antibiotics per patient | 3 (2-4) | 7 (4-7) | 3 (2-4) | 0.03 |
| Patients who died within 30 days of flap surgery were not divided into either of the groups. | | | | |
